# Revealing the complexity of meniscus microvasculature through 3D visualization and analysis

**DOI:** 10.1038/s41598-024-61497-2

**Published:** 2024-05-13

**Authors:** Federica Orellana, Alberto Grassi, Ruslan Hlushchuk, Peter Wahl, Katja M. Nuss, Antonia Neels, Stefano Zaffagnini, Annapaola Parrilli

**Affiliations:** 1 Center for X-Ray Analytics, Empa-Swiss Federal Laboratories for Materials Science and Technology, 8600 Dübendorf, Switzerland; 2 Department of Chemistry, University of Fribourg, 1700 Fribourg, Switzerland; 3 IRCCS-Rizzoli Orthopaedic Institute, 40136 Bologna, Italy; 4 Faculty of Medicine, University of Bern, 3012 Bern, Switzerland; 5 Cantonal Hospital Winterthur, 8401 Winterthur, Switzerland; 6 Vetsuisse Faculty, University of Zurich, 8057 Zurich, Switzerland

**Keywords:** Musculoskeletal system, Imaging techniques, Imaging techniques

## Abstract

Three-dimensional information is essential for a proper understanding of the healing potential of the menisci and their overall role in the knee joint. However, to date, the study of meniscal vascularity has relied primarily on two-dimensional imaging techniques. Here we present a method to elucidate the intricate 3D meniscal vascular network, revealing its spatial arrangement, connectivity and density. A polymerizing contrast agent was injected into the femoral artery of human cadaver legs, and the meniscal microvasculature was examined using micro-computed tomography at different levels of detail and resolution. The 3D vascular network was quantitatively assessed in a zone-base analysis using parameters such as diameter, length, tortuosity, and branching patterns. The results of this study revealed distinct vascular patterns within the meniscus, with the highest vascular volume found in the outer perimeniscal zone. Variations in vascular parameters were found between the different circumferential and radial meniscal zones. Moreover, through state-of-the-art 3D visualization using micro-CT, this study highlighted the importance of spatial resolution in accurately characterizing the vascular network. These findings, both from this study and from future research using this technique, improve our understanding of microvascular distribution, which may lead to improved therapeutic strategies.

## Introduction

The meniscus plays a critical role in maintaining the health and functionality of the knee joint^1,2^. One distinct feature of the meniscus is its vascular supply^3^. In 1982, Arnoczky and Warren investigated in detail the blood supply of the human meniscus by the use of histology and tissue clearing techniques^4^. They demonstrated that the meniscal blood supply is delivered by the lateral and medial genicular arteries, which form a perimeniscal capillary plexus within the synovial and capsular tissues of the knee joint. This plexus is oriented predominantly in a centripetal direction, with radial branches entering the meniscus from the outer portion. Further 2D radiographical^5^ and histological^6^ studies confirmed the results obtained by Arnoczky and Warren and indicated that the degree of vascular penetration within the meniscus is both age and zone dependent. In the adult, three differentially vascularized areas are present and the differentiation between red-red (RR), red-white (RW), and white-white (WW) zones is frequently used, describing decreasing blood supply^4,7^.

The regenerative potential of the meniscus depends on the presence of blood vessels. Indeed, tears in the vascularized area are able to promote the tissue healing due to the supply of oxygen, essential factors, and nutrients from the neighboring blood vessels; whereas damages in the avascular area are incapable of repair^8,9^. A comprehensive understanding of the microstructure of the meniscus is therefore of paramount importance, as meniscal injury is the most common lesion of the knee with an average annual incidence of 25–70/100,000^10–12^ and is a common cause of pain and disability. As awareness of the importance of the meniscus has increased, today's clinical management strategies prioritize conservative surgery whenever possible^13^. Especially in young patients, meniscal replacement or regeneration may help prevent early onset of OA or slow its progression. The optimal meniscal repair technique is adapted to the location of the lesion, as well as the anatomical and vascular characteristics. Neovascularization plays a fundamental role in meniscal healing, and clinically observed differences in repair are related to differences in vascular supply rather than cellular activity^14^.

Despite the importance of the subject, the work of Arnoczy and Warren^4^ remains practically the only point of reference, even though it is about 40 years old. In addition, although the RR, RW, and WW nomenclature is still commonly used, the International Society of Arthroscopy, Knee Surgery, and Orthopaedic Sports Medicine (ISAKOS) Meniscal Documentation Subcommittee has recommended against the use of these terms to classify meniscal tears, due to the variability in the vascular supply of the menisci^15^. The study of the meniscal microvasculature has the potential to be greatly enhanced by advances in modern techniques and 3D imaging. To date only few studies have tried to address the 3D complexity of the meniscal vascularization and its distribution. For example, the zone-dependent vascularization of healthy human menisci was qualitatively investigated and mapped using a combination of tissue clearing via the uDisco protocol and an immunolabeling approach^16^. Same approach was used to quantitatively analyze the blood supply to the meniscus in transgenic mice^17^. Other studies have used optical projection tomography (OPT), although they have focused on the fibrous microstructure of the meniscus rather than its vasculature, and on animal samples (bovine and lapine)^18,19^.

Among the numerous imaging techniques, X-ray micro-computed tomography (micro-CT) is a non-destructive method that allows for the investigation and analysis of the internal microstructure of materials and provides the ability to create 3D models of the analyzed samples^20–23^. The analysis of CT datasets permits to define several parameters and geometrical variables, which are relevant for describing the structure and properties of a sample^24,25^. In studies focused on meniscal analysis, micro-CT technology has been applied to a variety of investigations: geometry and dimensions^26–28^, calcification detection^29–31^, fibrous microstructure^11,32–36^, fluid flow regimes^37^, and contrast agent diffusion kinematics^38^. Despite this extensive application of micro-CT to various aspects of meniscal research, none of these studies have yet utilized this promising technique specifically to analyze vascularization within the meniscus.

The purpose of our study was therefore to investigate the feasibility of mapping and visualizing the microvasculature within the human meniscus using advanced 3D imaging techniques. In addition, we analyzed the 3D meniscal microvascular network's regional characteristics by defining different quantitative parameters, including vessel density, branching patterns, vascular mean diameter, length, and tortuosity.

## Materials and methods

### Sample preparation

**Figure 1:**
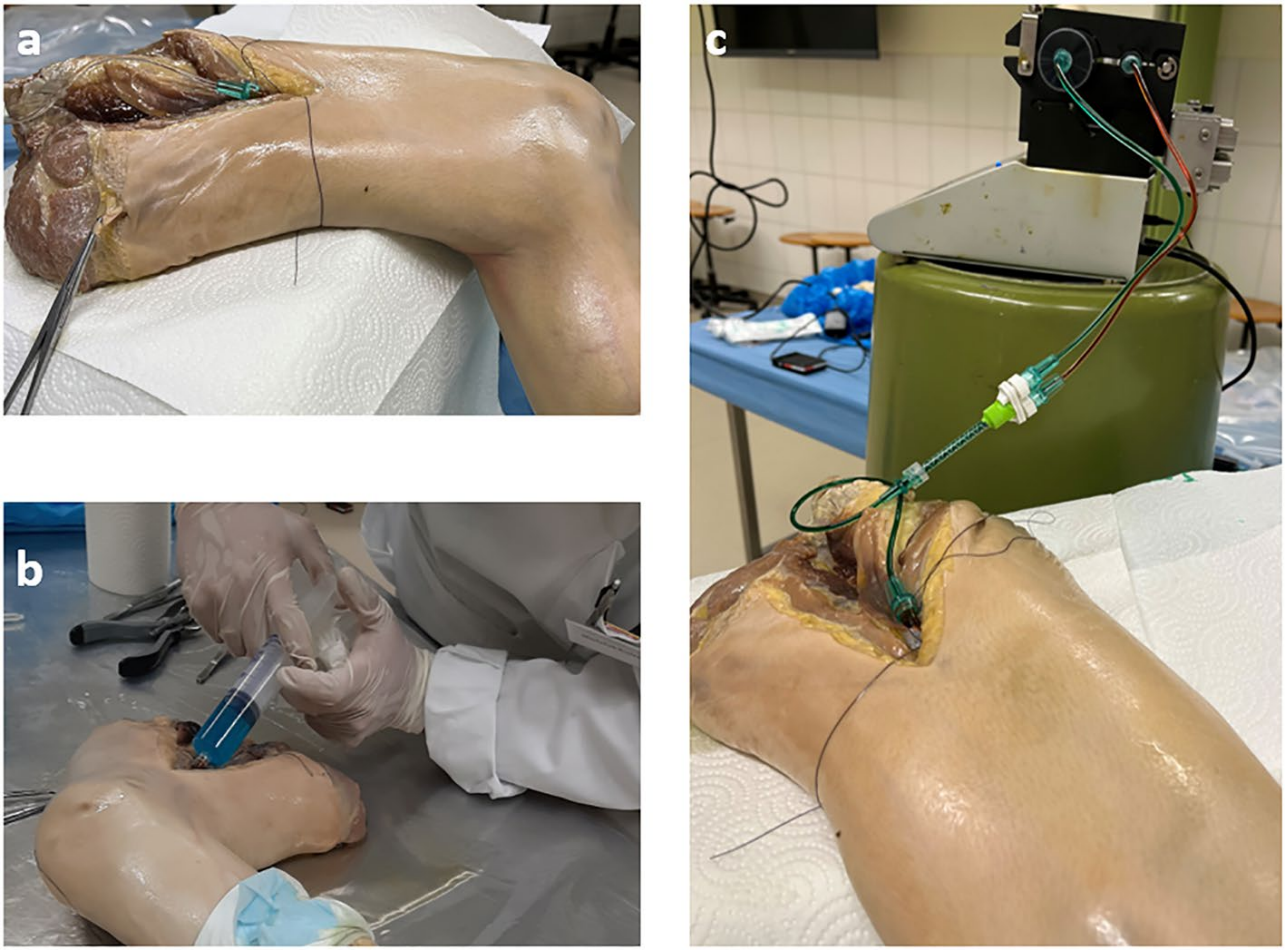
Contrast agent injection. (**a**) Cannulation of the femoral artery. (**b**) Intravascular perfusion with a silicone oil and a blue dye. (**c**) µAngiofil injection through the cannulated femoral artery.

All methods were performed in accordance with the relevant guidelines and regulations. Samples from three Thiel-fixated human cadaver legs (6 menisci), were provided by the Institute of Anatomy of the University of Bern. The donors were all male with a mean age of 75 (range, 62–86 years). Prior to perfusion, the polymer-based contrast agent µAngiofil was prepared according to the manufacturer's guidelines (Fumedica, Muri AG, Switzerland). Approximately 300 ml of the contrast agent was used for the perfusion of each leg. For the perfusion procedure, the femoral artery of the corresponding limb was cannulated (Fig. 1a). First, the corresponding limb was perfused with the low viscosity silicone oil (Bluesil, Elkem, Norway) with the addition of blue dye (Orasol Blue dye, BASF, Germany) to flush out the postmortem clots and restore the flow to the region of interest (Fig. 1b). Perfusion with oil was performed until the skin near the site of interest (knee joint) turned blue, indicating successful perfusion of the microvascular bed around the knee. Subsequently, the previously prepared µAngiofil was injected through the cannulated femoral artery at a volume rate of 20 ml/min (Fig. 1c). After the perfusion, the sample remained in a vertical position for 30 min until the complete polymerization of the contrast agent has occurred.

### Ethics declarations

The use of the human cadaveric material was performed according to the Swiss Federal Act on Research involving Human Beings n.810.30 (Human Research Act, HRA, of The Federal Assembly of the Swiss Confederation) of 30 September 2011 and the Guidelines of the Swiss Academy of Medical Sciences, updated 2014. Donors have formally agreed the use of body parts for research by signing the informed consent forms. The experimental protocols have been approved by the Management Board of the Institute of Anatomy, University of Bern.

### Analysis classification system

**Figure 2:**
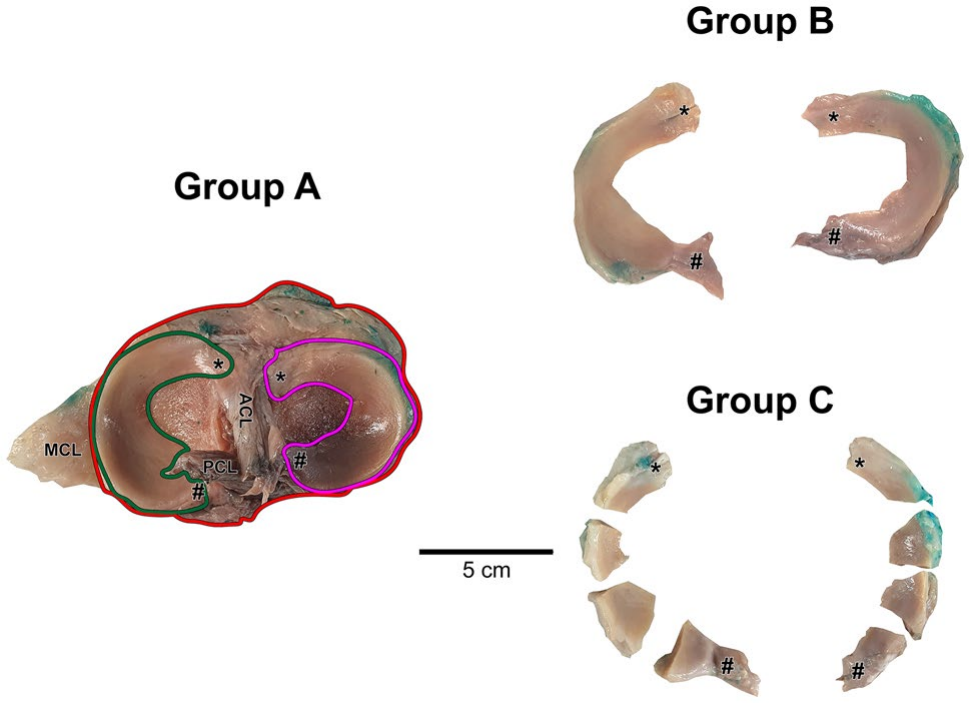
Sample preparation. Group A: axial view of the human tibial plateau showing the semicircular shapes of the menisci and their relationship to the cruciate ligaments. Group B: entire human medial and lateral menisci highlighting the anterior (*) and posterior (#) roots. Group C: human menisci divided into four portions. The tibia is outlined in red, the lateral meniscus in pink, and the medial meniscus in green. TL, transverse ligament; MCL, medial collateral ligament; ACL, anterior cruciate ligament; PCL, posterior cruciate ligament. Scale bar 5 cm.

Three analysis groups were defined and named in alphabetical order from the letter A to C (Fig. 2). First, the knee was excised with a cut approximately 10 cm above and below the tibiofemoral joint. The specimens were gradually reduced in size in order to increase the applicable resolution for the micro-CT analysis. The outer layer of skin, the patella, the fibula, all muscles, tendons, and ligaments, except for the medial collateral ligament (MCL), the anterior cruciate ligament (ACL) and the posterior cruciate ligament (PCL), the anterior and posterior meniscal root attachments, and the transverse ligament (TL), were removed. Afterwards, the femur was removed by cutting the MCL and cruciate ligaments respectively a few centimeters above the upper edge of the medial meniscus and at the level of their femoral attachment in the intercondylar fossa. The samples obtained in this way belong to group A and consist of the tibia, the menisci, the ACL, the PCL, the TL, and the anterior and posterior meniscal root attachments.

The menisci were then carefully dissected from the rest of the joint capsule and cut at the level of the anterior and posterior roots (group B). Anteriorly, the menisci were separated by incision of the TL and the medial meniscus was further isolated from the MCL. Additionally, the menisci were subdivided into four radial portions cut at angles of 45°, 90° and 135° relative to the sagittal plane (Fig. 2) that belong to the classification group C.

### Micro-CT imaging

Micro-CT analysis was carried out using an EasyTom XL Ultra 230–160 micro/nano-CT scanner (RX Solutions, Chavanod, France). The specimens were analyzed starting from the entire joint, and then scanned once gradually reduced in size to increase the applicable resolution accordingly *(from group A to C)*. Each CT dataset generated was compared and combined with its group A reference sample, to accurately maintain anatomical and morphological references. All setting parameters used for the scanning and reconstruction are described in detail in Table 1. Briefly, the scanner operated at 100 kV and 100 μA for group A, and at 70 kV and 70 μA for groups B and C. All the samples were scanned over 360° with a rotation step of 0.25° and a frame average of 10. The nominal resolution was set to 60 μm, 30 μm, and 15 μm voxel (Vx) size for groups A, B and C, low, medium, and high resolution, respectively. All the CT datasets were reconstructed using the filtered back-projection algorithm, a small ring artefact reduction and a 65% Hann window function.

**Table 1:** Overview of the parameters used for micro-CT analysis and reconstruction accordingly to the sample group.

Sample	Anatomical parts	CT analysis settings	Reconstruction parameters
Group A (low resolution)	Tibia, menisci, cruciate ligaments	100 kV, 100 μA Rotation step 0.25 Frame average 10 60 μm Vx size	Filtered backprojection (tomography) Format: 16 bit Tiff Noise filter: 65% Hann 5 pixels ring filter
Group B (medium resolution)	Intact menisci	70 kV, 70 μA Rotation step 0.25 Frame average 10 30 μm Vx size	Filtered backprojection (tomography) Format: 16 bit Tiff Noise filter: 65% Hann 0 pixels ring filter
Group C (high resolution)	Partitioned menisci	70 kV, 70 μA Rotation step 0.25 Frame average 10 15 μm Vx size	Filtered backprojection (tomography) Format: 16 bit Tiff Noise filter: 65% Hann 10 pixels ring filter

### Segmentation of the vascular network

The generated CT datasets were analyzed using the open-source image processing package Fiji^39^ and the software application Avizo (Thermo Fisher Scientific, MA, USA). First, the image background was removed. The meniscal vascular network was then segmented combining the Max Entropy algorithm^40^ with the white top-hat operation.

In the Max Entropy algorithm, the optimal threshold t* is defined as the gray level which maximizes two measures of the a posteriori information associated with the black and white pixels after thresholding, *H*_*b*_*(t)* and *H*_*w*_*(t)*^41^, Eq. (1):

1 $$t^{*} = {\text{ArgMax}}\left\{ {H_{b} \left( t \right) + H_{b} \left( t \right)} \right\}$$

The white top-hat operator was used for light objects on a dark background and is defined as the difference between the original grayscale input image and the opening operation^42^, Eq. (2):

2 $$T_{{{\text{hat}}}} \left( f \right) = f - \left( {f \circ b} \right)$$

In this combined process of segmentation, the Max Entropy algorithm permits to identify the most accurate grayscale range for the extraction of the main vascular network formed by large and high attenuating blood vessels, whereas the white top-hat method allows to select details, the minor vessels, which are smaller than the structuring element and brighter than the surroundings.

### 3D quantitative analysis of the vascular network

The centerlines of the filamentous structures were extracted from the segmented image using the Auto Skeleton (Avizo 3D) module^43^ to analyze individual vessel branches. The module first computes a distance map of the segmented image, then performs a thinning of the binary image so that a string of connected voxels remains. The voxel skeleton is then organized as a graph consisting of branches or endpoints of the network, called "nodes", and curved lines connecting the nodes, called "segments". Depending on the number of incident segments, nodes are classified as terminal if they have one incident segment, or as branching if they have three or more incident segments. The three-dimensional (3D) course of a segment is defined by a sequence of points in 3D space, and a set of segments connected by nodes is called a "graph".

The vascular network was assessed by evaluating important vascular parameters, including segment curved length, diameter, and tortuosity. Segment length is defined as the sum of lengths between points defining the vessel segment. Segment diameter is calculated as the average value of local vessel diameter over all the points defining the segment, and tortuosity as the ratio between the effective distance and the shortest distance between the two branch points associated to the segment. All the parameters were calculated for meniscus scans across the three groups—Group A (low resolution), Group B (medium resolution), and Group C (high resolution)—to evaluate the effect of micro-CT image resolution on the analysis, and for anatomical areas. Each meniscus was therefore divided into four radial areas (anterior, mid-anterior, mid-posterior, and posterior), each comprising one fourth of the meniscus, and four circumferential zones defined by the Cooper zone classification system^44^ (perimeniscal (PM), zone 1 (RR) outer third, zone 2 (RW) middle third, and zone 3 (WW) inner third). The perimeniscal zone was delineated based on its different appearance and density in the micro-CT images and the geometry of the meniscus, while the remaining circumferential zones (1, 2, and 3 zones) were systematically divided into thirds, each representing 33% of the radius from the identified periphery toward the inner regions. The meniscal vascular contribution for the circumferential and radial zones was determined as the ratio between the voxels identified as vessels for each zone and the total number of voxels associated with the vascular network.

### Statistical analysis

All data are presented as mean ± standard deviation unless otherwise stated. The standard error of the mean (SEM) of the parameters number of segment, number of nodes, and branching nodes was calculated pooling first data across the graphs that resulted in the image analysis of the vessels. Single Factor Anova and Turkey-Kramer's Test were performed for multiple comparisons. Statistical significance was set at *P* < 0.05.

## Results

### Vessel segmentation

**Figure 3:**
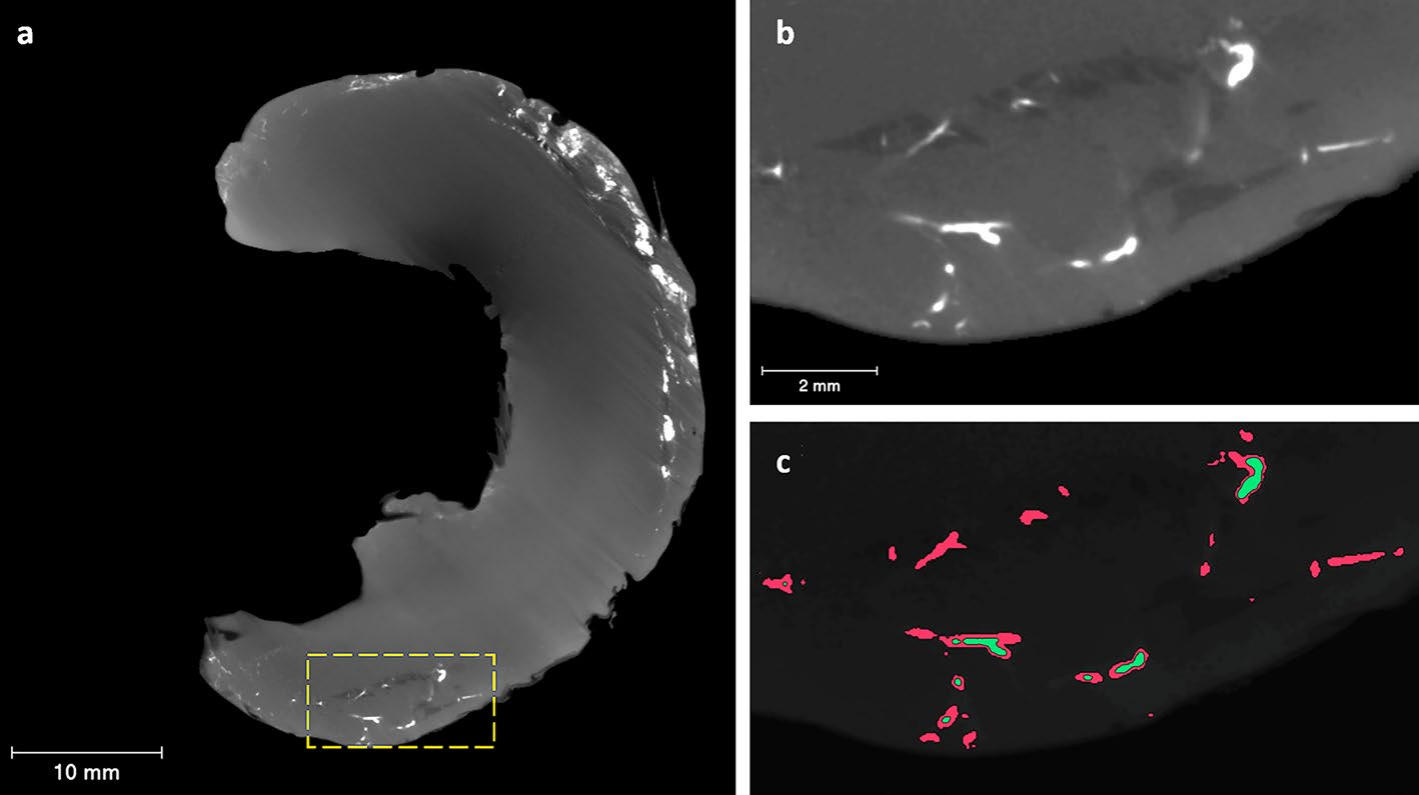
Human meniscus micro-CT 2D sections. (**a**) Axial micro-CT image of the lateral meniscus, showing the vascular network in white. (**b**) The zoom-in area of the yellow dotted-line box that focuses on a vascularized region. (**c**) Binary image of the zoom-in area obtained by automatic segmentation of blood vessels. Green is the segmentation result using the Max Entropy algorithm and red color represent the result after the white top-hat operation. Scale bars: 10 mm (**a**), 2 mm (**b**).

To segment the blood vessels, we used the Max Entropy algorithm, which maximizes the inter-class variance in an image^45^, and then combined it with the top-hat operation, since the use of one-step segmentation method was not sufficient to segment all the blood vessels. In this way, the Max Entropy algorithm permitted to identify the most accurate grayscale range for the extraction of the main vascular network formed by large and high attenuating blood vessels, whereas the white top-hat method allowed to select details, the minor vessels, which are smaller than the structuring element and brighter than the surroundings (Fig. 3).

### 3D imaging of the meniscal vascular network

**Figure 4:**
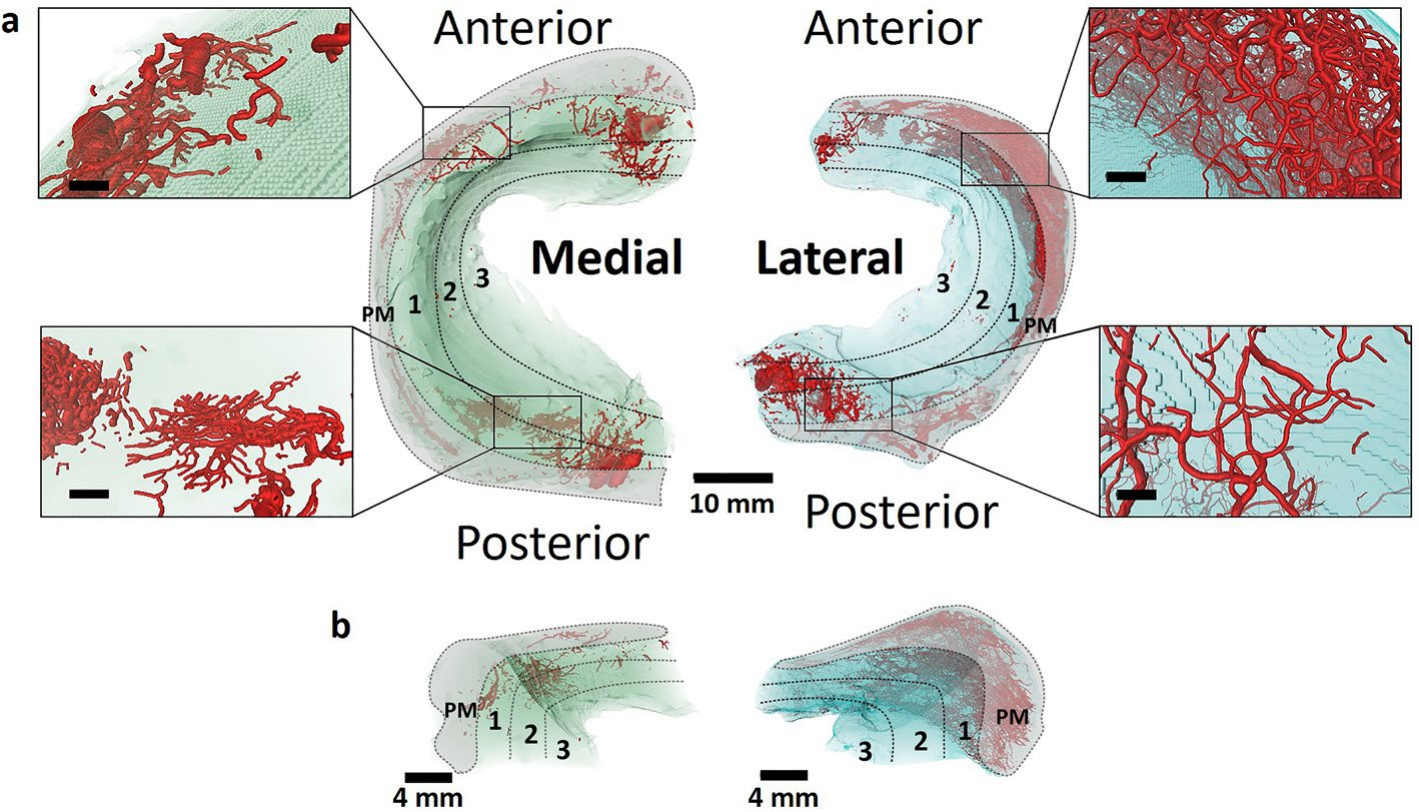
Vasculature of the human medial and lateral menisci visualized by micro-CT. (**a**) Axial 3D view of the medial and lateral menisci with the corresponding circumferential areas. Zoomed areas with high vascularization are displayed at the exterior of each meniscus. Scale bars: 10 mm (entire meniscus), 1.5 mm (zoom-in areas). (**b**) Radial 3D view of the medial and lateral menisci with the corresponding circumferential areas. Scale bars: 4 mm. Medial meniscus is outlined in green, lateral meniscus in light blue, and vascular network in red with diameter-proportional blood vessel tubes. PM = Perimeniscal Zone, Cooper Zone 1 (RR = red-red), Cooper Zone 2 (RW = red-white), Cooper Zone 3 (WW = white-white). Voxel size 30 μm.

Micro-CT analysis in combination with the injection of the polymerizing contrast agent μAngiofil allowed imaging of the meniscus and its 3D vascular network. Following the automatic segmentation approach described above, the meniscal volumes were modelled using a volume rendering technique that allowed visualization of the 3D morphology and architecture of the medial and lateral menisci and their respective vascular networks (Fig. 4).

**Figure 5:**
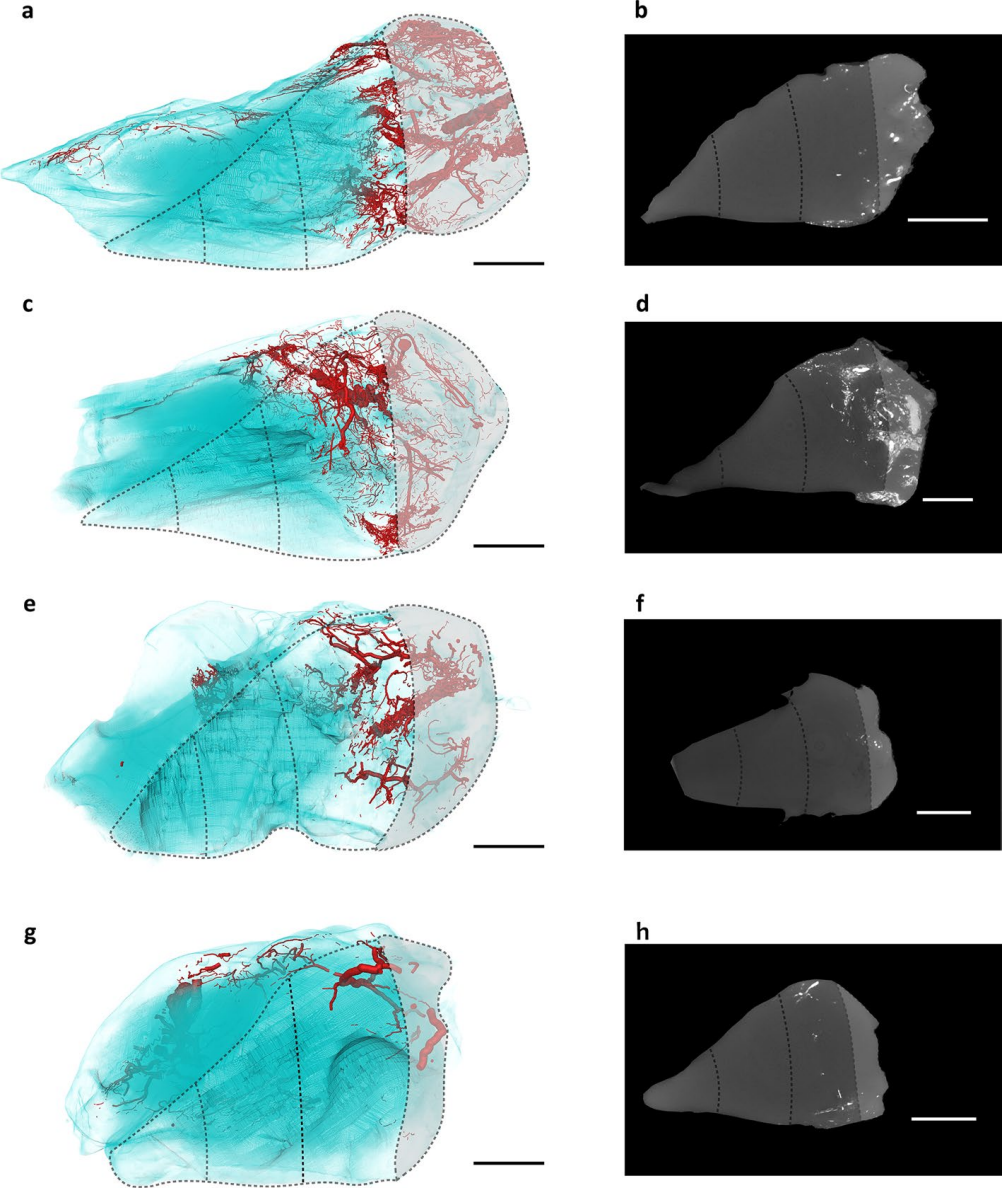
Vasculature of the radial portions of the lateral meniscus visualized by micro-CT. (**a**,**c**,**e**,**g**) Radial 3D views of the anterior, mid-anterior, mid-posterior, and posterior portions, respectively, with the corresponding circumferential areas. Lateral meniscus is outlined in light blue and vascular network in red with diameter-proportional blood vessel tubes. (**b**,**d**,**f**,**h**) Cross-sectional 2D images of the anterior, mid-anterior, mid-posterior, and posterior portions, respectively, with the corresponding circumferential areas. Scale bars: 4 mm. Voxel size 15 μm.

In addition, 3D macrosections of the radial portions (thickness ≈ 1 cm) can be visualized and compared with 2D micro-CT sections (Fig. 5), highlighting the power of 3D information.

**Figure 6:**
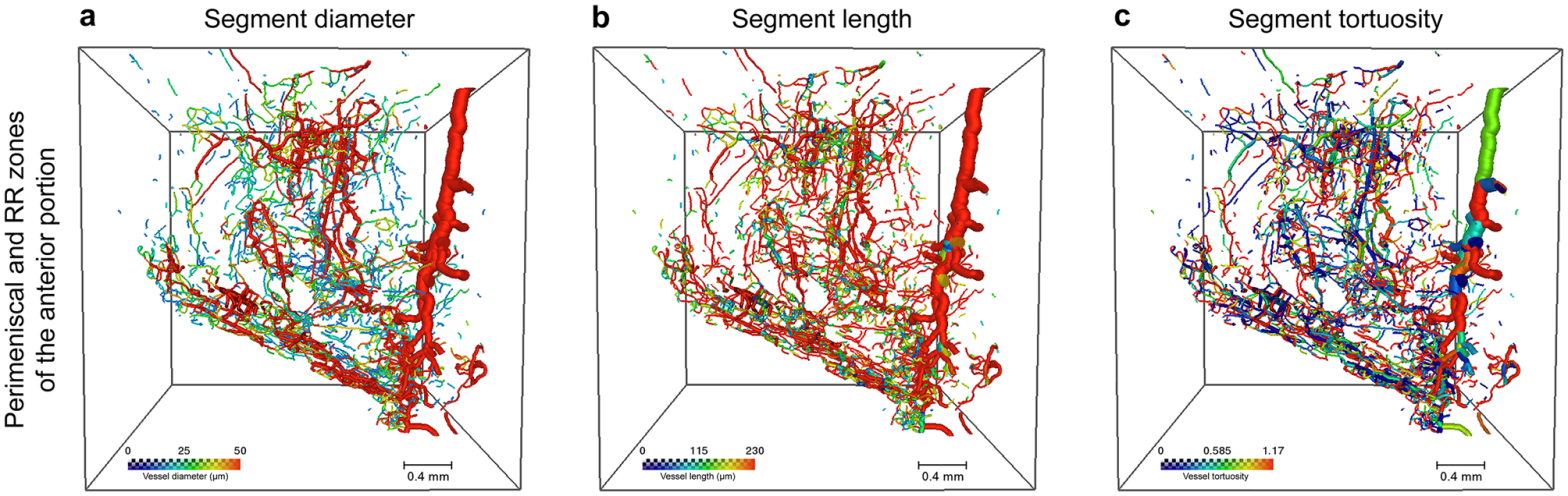
Vasculature of the anterior radial portion of the lateral meniscus visualized by micro-CT. Axial view of the vascular network displayed with diameter-proportional blood vessel tubes. Vessels are colour-coded according to either their diameter (**a**), length (**b**) or tortuosity (**c**). PM = Perimeniscal Zone, Cooper Zone 1 (RR = red-red). Voxel size 15 μm. Scale bars 0.4 mm.

In the high resolution scans, a more detailed analysis of the vascular network was performed. It was possible to visualize and characterize the vascular segments according to their diameter, length and tortuosity using color-coded images (Fig. 6).

### Meniscal vascular volume contribution in the respective circumferential portions

**Figure 7:**
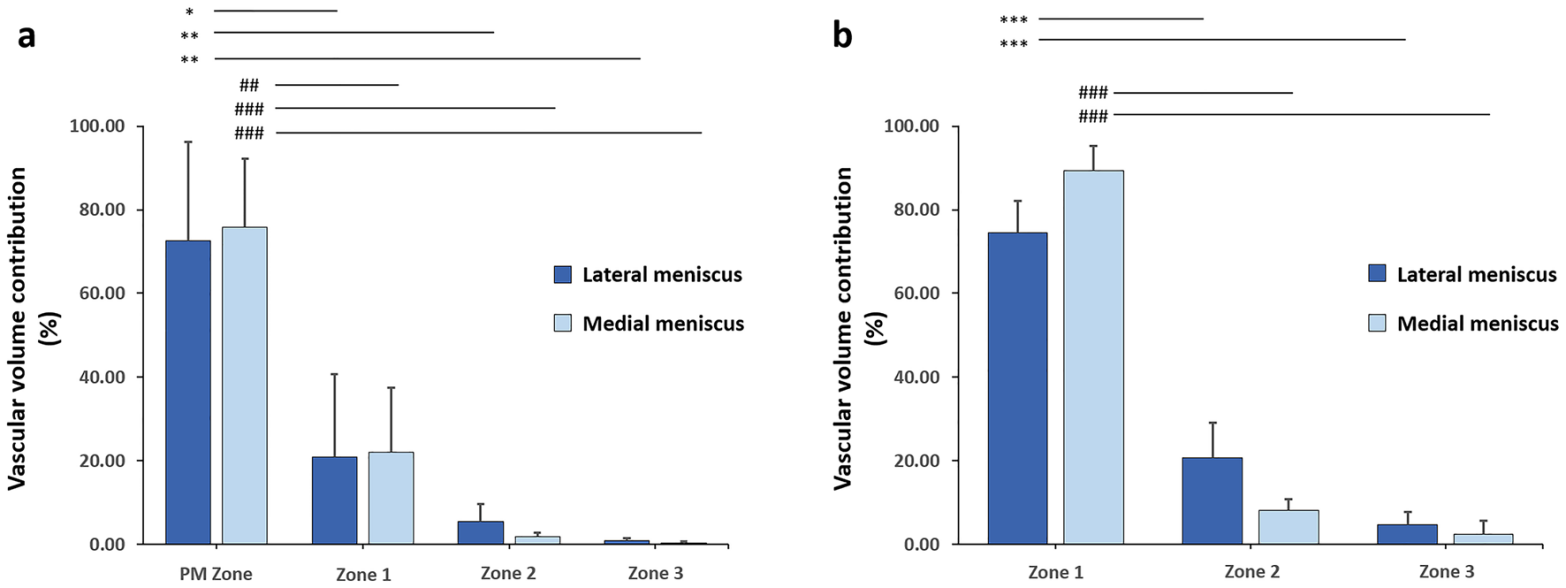
Meniscal vascular volume contribution over the circumferential portions. (**a**) Vascular volume contribution in all zones. (**b**) Vascular volume contribution without the perimeniscal zone. PM = Perimeniscal Zone, Cooper Zone 1 (RR = red-red), Cooper Zone 2 (RW = red-white), Cooper Zone 3 (WW = white-white). *0.01 < *P* < 0.05; **/^##^0.001 < *P* < 0.01; ***/^###^*P* < 0.001. The analysis referred to group B-medium resolution scans (N = 3 for each circumferential zone).

The perimeniscal zone showed the highest vascular volume contribution, with more than 72% of the blood vessels in the lateral and medial menisci located in this area. The meniscal vascular density in the lateral meniscus was higher in perimeniscal (PM) zone than in Cooper zone 1 (*P* = 0.015), zone 2 (*P* = 0.003), and zone 3 (*P* = 0.002, Fig. 7a). Similarly to the lateral meniscus, the meniscal vascular density in the medial meniscus was higher in perimeniscal zone than in the remaining circumferential zones (PM zone vs. zone 1 *P* = 0.002, PM zone vs. zone 2 *P* < 0.001, PM zone vs. zone 3 *P* < 0.001) (Fig. 7a). Without the perimeniscal area, zone 1 displayed the highest vascular volume (lateral meniscus: zone 1 vs. zone 2 *P* < 0.001, zone 1 vs. zone 3 *P* < 0.001; medial meniscus: zone 1 vs. zone 2 *P* < 0.001, zone 1 vs. zone 3 *P* < 0.001) (Fig. 7b). The contribution of zone 3 to the overall meniscal vasculature is less than 5% in the lateral meniscus and 2.5% in the medial meniscus (Fig. 7b).

### Meniscal vascular volume contribution in the respective radial portions

**Figure 8:**
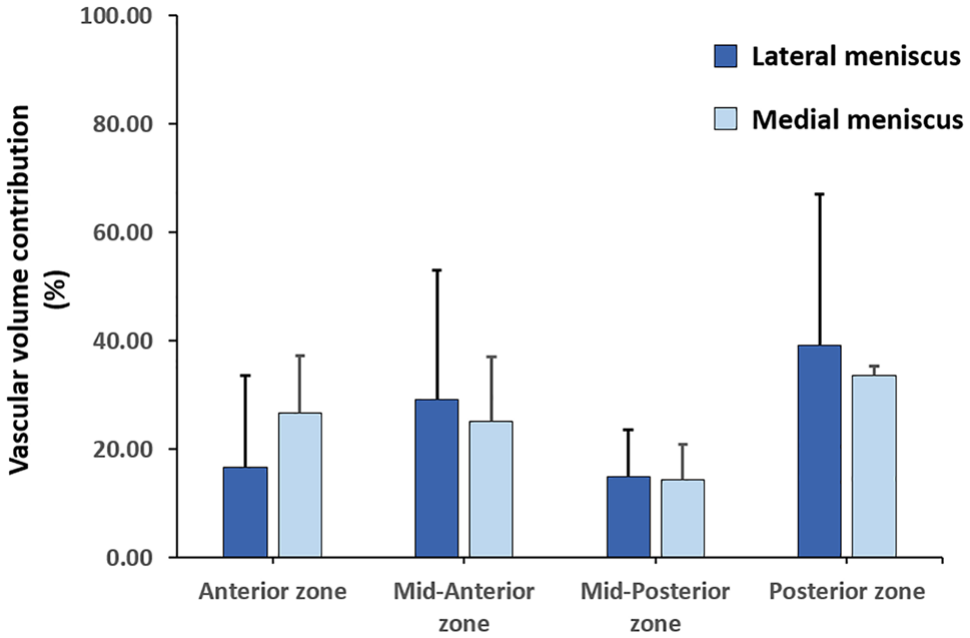
Meniscal vascular volume contribution over the radial portions. The analysis referred to group C–high resolution scans (N = 3 for each radial zone).

The majority of blood vessels in the lateral meniscus were found to be located in the mid-anterior and posterior zones, accounting for 68% of the total vessel volume. The remaining vessel volume was distributed in the anterior and mid-posterior zones, with 17% and 15% of the overall vascular volume found in these areas, respectively (Fig. 8). In the medial meniscus, the anterior, mid-anterior, and posterior regions had the highest volume of blood vessels, accounting for more than 80% of the total vessel volume. Similarly to the lateral meniscus, the mid-posterior portion showed the lowest contribution to the overall meniscal vasculature (Fig. 8).

### Vascular topology and connectivity

**Figure 9:**
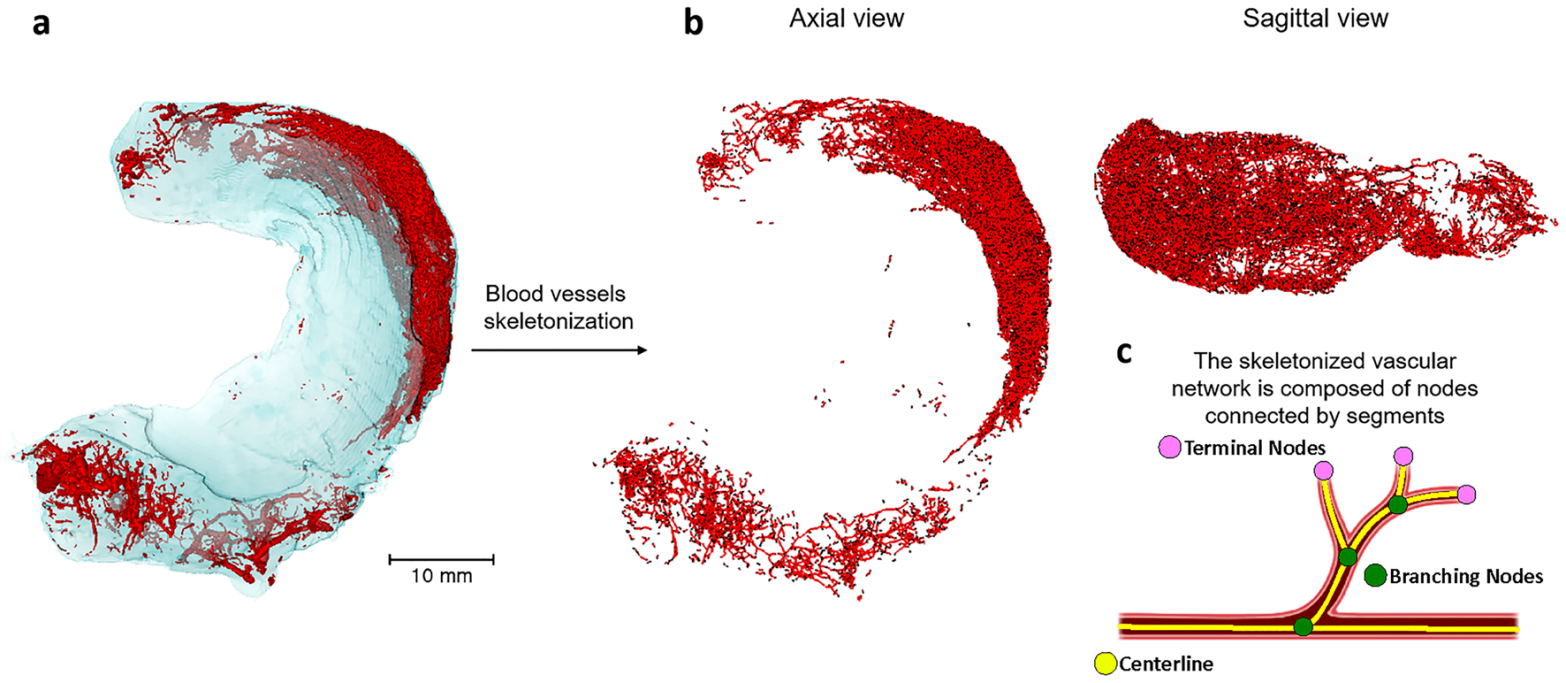
Visualization of the image segmentation and skeleton tracing process. (**a**) 3D volume rendering of the lateral meniscus after vascular segmentation. (**b**) Axial and sagittal views of blood vessels after skeletonization. (**c**) Organization of the skeletonized vascular network. Scale bar 10 mm.

The vascular network was segmented for all the anatomical groups. Identification of blood vessels within the tissue allowed to measure the vascular volume in the lateral and medial menisci at each stage of sample processing. After the skeletonization, each individual vessel segment was distinguished and the vascular network was assessed by evaluating key vascular parameters, including total length, mean diameter, number of segments, and nodes (Fig. 9).

The analysis performed at higher spatial resolutions (groups B–medium resolution and C-high resolution scans) allowed the identification of a greater number of vascular segments and nodes than in group A-low resolution scan—in the lateral and medial menisci (lateral meniscus no. of segments: *P* = 0.016; no. of nodes: *P* < 0.001) (medial meniscus no. of segments: *P* < 0.001; no. of nodes: *P* < 0.001). The vascular segments of groups B–medium resolution and C-high resolution formed a vascular network with a greater total length than in group A-low resolution scan. The higher resolution analysis enabled the detection of smaller vessels, hence the average diameter value is lower in groups B-medium resolution and C-high resolution than in group A-low resolution. The number of segments, nodes, and the total length of the medial meniscus were also statistically higher in group C-high resolution than in group B-medium resolution (no. of segments:* P* < 0.001, no. of nodes: *P* < 0.001, total length: *P* < 0.001) (Table 2).

**Table 2:** Quantitative vascular parameters obtained analyzing the lateral and medial menisci for each resolution scan group.

	Lateral meniscus			Medial meniscus		
	Group A-low resolution	Group B-medium resolution	Group C-high resolution	Group A-low resolution	Group B-medium resolution	Group C-high resolution
Total volume (mm^3^)	106.26 ± 21.69	120.59 ± 11.40	228.32 ± 18.93	37.29 ± 5.13	94.39 ± 9.24	133.85 ± 11.54
No. of segments	816 ± 138	13,700 ± 1884	*22,663 ± 1274	559 ± 70	3752 ± 319	***25,962 ± 1263^###^
Total length (mm)	700.69 ± 123.47	4567.81 ± 558.31	5904.78 ± 355.89	464.91 ± 57.58	1817.24 ± 156.77	***5591.97 ± 219.68^###^
Mean diameter (μm)	172.86 ± 89.44	80.42 ± 12.73	72.81 ± 8.33	135.24 ± 29.19	92.71 ± 12.85	66.09 ± 8.88
No. of nodes	644 ± 102	10,592 ± 1337	***18,816 ± 904	581 ± 76	3477 ± 295	***21,417 ± 979^###^

Data are shown as mean ± SEM. *0.01 < *P* < 0.05; ***/^###^*P* < 0.001 (N = 3 for each group).

**Figure 10:**
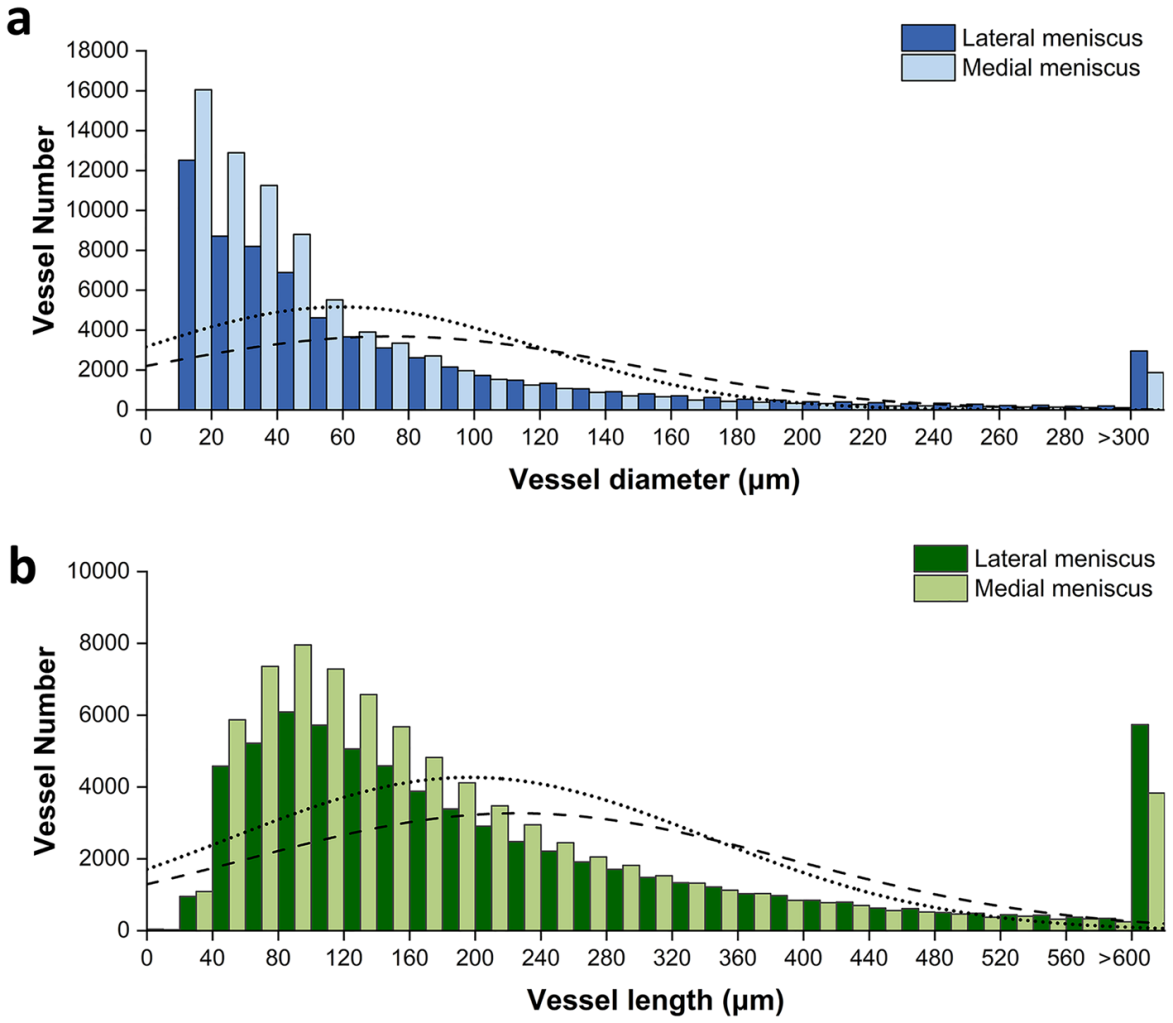
Vessel size distribution in the lateral and medial menisci. (**a**) Vessel diameter distribution. (**b**) Vessel length distribution. The analysis referred to group C-high resolution scan. Dashed and dotted lines indicate the average frequency of vessel size for the lateral and medial menisci respectively.

Figure 10 shows the vessel size distribution of the lateral and medial menisci of group C-high resolution scan. The majority of blood vessels (Vessel Number > 1000) in the lateral and medial menisci have a diameter ranging from 15 to 135 μm. The dashed and dotted lines, representing the average frequency of vessel diameter, indicate that the average diameter values are slightly different between the lateral (65–75 μm) and medial (45–65 μm) meniscus (Fig. 10a). The vascular length of the lateral and medial meniscus ranged from 45 to 370 μm (Vessel Number > 1,000) with a mean value close to 250 μm and 220 μm respectively (Fig. 10b).

Vascular parameters, including number of segments, nodes, and branching nodes, mean diameter and length, total length, and tortuosity, were calculated for each circumferential (Table 3) and radial (Table 4) meniscal zone. These analyses revealed the intricate architecture of the vascular system within the meniscus, and showed a vascular pattern that was dependent on both the circumferential and radial portions examined. In particular, the vascular segments of perimeniscal zone had a different diameter compared to the other circumferential zones of both menisci (lateral meniscus – mean diameter: PM zone vs. Cooper zone 1 *P* < 0.001, PM zone vs. zone 2 *P* < 0.001, PM zone vs. zone 3 *P* < 0.001, zone 1 vs. zone 2 *P* < 0.001, zone 1 vs. zone 3 *P* < 0.001, zone 2 vs. zone 3 *P* < 0.001) (medial meniscus – mean diameter: PM zone vs. zone 1 *P* < 0.001, PM zone vs. zone 2 *P* < 0.001, PM zone vs. zone 3 *P* < 0.001). In the lateral meniscus, the mean length of blood vessels in perimeniscal zone had a different value compared to the other circumferential zones (mean length: PM zone vs. zone 1 *P* < 0.001, PM zone vs. zone 2 *P* < 0.001, PM zone vs. zone 3 *P* < 0.001). The tortuosity of blood vessels in zone 1 of the lateral meniscus was different from that observed in zone 3 (zone 1 vs. zone 3 *P* = 0.015) (Table 3).

**Table 3:** Quantitative vascular parameters obtained analyzing the circumferential portions of the lateral and medial menisci.

	Lateral meniscus				Medial meniscus			
	PM zone	Zone 1 (RR)	Zone 2 (RW)	Zone 3 (WW)	PM zone	Zone 1 (RR)	Zone 2 (RW)	Zone 3 (WW)
No. of segments	8762 ± 13	2900 ± 3	866 ± 3***	82 ± 1***	1760 ± 1	1134 ± 3	112 ± 1	22 ± 0.39
Mean diameter (μm)	76.60 ± 0.49	***87.86 ± 1.09	98.43 ± 2.56^###^	^%%%^56.51 ± 1.52^###^	130.98 ± 1.72	***113.48 ± 2.04	***83.90 ± 2.91	***49.07 ± 2.81
Mean length (mm)	0.318 ± 0.001	0.374 ± 0.003***	0.359 ± 0.006***	0.361 ± 0.012***	0.49 ± 0.005	0.47 ± 0.007	0.47 ± 0.021	0.52 ± 0.063
Total length (mm)	3099.34 ± 3.78	1084.47 ± 0.93	311.14 ± 1.03	72.84 ± 0.40	1215.55 ± 0.63	537.57 ± 1.63	52.78 ± 0.67	11.34 ± 0.23
Tortuosity	1.12 ± 0.002	1.15 ± 0.005	1.14 ± 0.009	1.18 ± 0.023^#^	1.16 ± 0.006	1.15 ± 0.007	1.14 ± 0.019	1.13 ± 0.052
No. of nodes	7154 ± 9	2461 ± 2	748 ± 2	229 ± 0.9	2316 ± 1	1016 ± 2	116 ± 1	30 ± 0.30
Branching nodes	5072 ± 7	1415 ± 1	405 ± 2	75 ± 1	1167 ± 1	549 ± 2	48 ± 1	6 ± 0.23

PM = Perimeniscal Zone, Cooper Zone 1 (RR = red-red), Cooper Zone 2 (RW = red-white), Cooper Zone 3 (WW = white-white). Data are shown as mean ± SEM. ^#^0.01 < *P* < 0.05; ***/^###^/^%%%^*P* < 0.001 (N = 3 for each circumferential zone).

**Table 4:** Quantitative vascular parameters obtained analyzing the radial portions of the lateral and medial menisci.

	Lateral meniscus				Medial meniscus			
	Anterior	Mid-anterior	Mid-posterior	Posterior	Anterior	Mid-anterior	Mid-posterior	Posterior
No. of segments	3663 ± 1	4988 ± 1	4349 ± 5	*9663 ± 9	6075 ± 3	5632 ± 7	3975 ± 2	10,280 ± 4
Mean diameter (μm)	51.75 ± 0.52	***79.95 ± 0.49	***83.06 ± 0.85	***^%%%^95.03 ± 0.71^###^	56.51 ± 0.42	***75.06 ± 0.30	***40.50 ± 0.44	***^%%%^66.86 ± 0.65^###^
Mean length (mm)	0.232 ± 0.002	0.227 ± 0.003	0.230 ± 0.003	^%%%^***0.262 ± 0.002^###^	0.222 ± 0.002	0.218 ± 0.002	***0.208 ± 0.003^#^	%%0.219 ± 0.002
Total length (mm)	1.25 ± 0.23	1.30 ± 0.31	2.65 ± 1.30	**4.17 ± 2.51	1.95 ± 0.71	2.63 ± 1.34	1.32 ± 0.47	1.20 ± 0.78
Tortuosity	1.13 ± 0.004	1.12 ± 0.004	***1.09 ± 0.004^###^	***1.10 ± 0.003^%%%^	1.12 ± 0.003	1.13 ± 0.003	1.15 ± 0.004^#^	^%%%^**1.15 ± 0.003
No. of nodes	3516 ± 1	4837 ± 1	3401 ± 3	**7062 ± 6	5079 ± 2	4356 ± 4	3479 ± 2	8502 ± 3
Branching nodes	1641 ± 1	2166 ± 0.5	2119 ± 3	*4735 ± 5	2987 ± 2	2824 ± 4	1865 ± 1	5033 ± 2

Data are shown as mean ± SEM. */^#^0.01 < *P* < 0.05; **/^##^/^%%^0.001 < *P* < 0.01; ***/^###^/^%%%^*P* < 0.001; (N = 3 for each radial zone).

In the radial portions, the vascular network showed a zone-dependent structure and organization (Table 4). The vascular diameter varies in all radial zones of the meniscus with the exception of the comparison between the mid-anterior and mid-posterior portions in the lateral meniscus (*P* < 0.001 for all comparisons). The mean length is different in the posterior part compared to the other portions of the lateral meniscus (*P* < 0.001 for all comparisons), whereas in the medial meniscus the length is different in the mid-posterior portion compared to all the other portions (ant. vs. mid-post *P* < 0.001, mid-ant. vs. mid-post. *P* = 0.017, mid-post. vs. post. *P* = 0.008). In addition, in the lateral meniscus, the tortuosity values are different in the comparisons between the anterior and mid-posterior (*P* < 0.001), anterior vs. posterior (*P* < 0.001), mid-anterior vs. mid-posterior (*P* < 0.001), and mid-posterior vs. posterior (*P* < 0.001) portions. In the medial meniscus, the tortuosity values were found to be different in the comparison between the anterior and posterior (*P* = 0.004), mid-anterior and mid-posterior (*P* = 0.025), mid-posterior and posterior (*P* < 0.001) portions. The number of nodes and branching nodes in the lateral meniscus is higher in the posterior than in the anterior portion (no. of nodes: *P* = 0.002, no. of branching nodes: *P* = 0.039).

## Discussion

In this study, the potential in conducting a comprehensive analysis of the microvasculature within human menisci was investigated employing advanced 3D visualization techniques. Through micro-CT imaging, we obtained high-resolution 3D representations of the intricate network of blood vessels within the meniscus, revealing the spatial arrangement, connectivity and density of the microvasculature.

The use of micro-CT ensured the mapping of blood vessels in a non-destructive way, preserving the structural complexity of the vascular network within the tissue. In this way, the menisci were analyzed at different levels of spatial resolution for each stage of sample processing. Variations were observed between the groups analyzed at different spatial resolutions (groups A-low resolution, B-medium resolution, and C-high resolution). In particular, the analysis performed at higher spatial resolutions (30 and 15 μm voxel size) allowed the identification of a greater number of vascular segments and nodes than in group A-low resolution (60 μm voxel size). These vascular segments have a smaller average diameter and they form a vascular network with a greater total length than group scanned at low resolution. These different findings in vascular parameters indicate the importance of spatial resolution for a detailed, accurate, and precise study of the vascular network.

Considering the overall volume contribution of blood vessels in the 4 circumferential zones of the lateral and medial meniscus (perimeniscal, zone 1-RR, zone 2-RW, and zone 3-WW), a higher vascular value was found in the perimeniscal zone compared to all other zones. While, not considering the perimeniscal area, most of the blood vessels were located in the zone 1 as expected given literature data on tissues from the same patient age^6^. Several studies have focused on the vascular architecture of the different areas of the meniscus, most of which have examined the circumferential areas. First, Arnoczky and Warren^4^, using histological and tissue clearing techniques, identified the presence of a perimeniscal capillary plexus originating in the capsular and synovial tissues of the joint and penetrating approximately 10–25% of the periphery of the meniscus. The rest of the tissue appeared mostly avascular (zones 2 and 3). Similarly, the results of our work confirm that the majority of the blood vessels are located in the outer area, at the junction to the capsule, which has more than 72% of the total blood vessels. Moreover, with respect to the radial portions, the mid-posterior portion showed a reduced vascular density, a result consistent with the findings of Arnoczky and Warren^4^. Through the immunohistochemical analysis of serial sections of menisci harvested from adults with bone tumor or OA, Michel et al*.*^6^ recently validated that the highest vascular density is present in the perimeniscal zone while no blood vessels were found in zone 2 and zone 3 after adolescence. Contrary to Michel et al*.*^6^, in our study some vascular vessels were found in the inner areas of the tissue, which might be attributed to the comprehensive 3D analysis of the entire meniscus by micro-CT imaging or because we analyzed samples from healthy donors, even if they are elderly. In fact, similarly to our study, Chahla et al*.*^16^ characterized the vascularity of menisci collected from healthy donors and found the presence of blood vessels in all areas of the meniscus using 3D immunofluorescence imaging analysis, including the area previously defined as avascular (zone 3-WW).

In addition to micro-CT analysis at different resolutions and the analysis of the vascular volume contribution, the main morphological and topological vascular parameters, including total length, mean diameter, number of segments and nodes, were calculated for all the circumferential and radial meniscal zones. In the lateral and medial meniscus, the outer perimeniscal region is the zone that exhibits the greatest dissimilarity compared to the other surrounding zones. Investigating morphological and topological vascular parameters is crucial for understanding the intricate processes of angiogenesis and collateral vessel growth, which are essential for proper blood vessel formation and the effective delivery of oxygen and nutrients to developing organs in the field of orthopedics. Vascular parameters, such as the density, length, diameter and tortuosity of blood vessels, are closely related to the dynamics of blood flow, remodeling of the vascular network, and transport of nutrients and oxygen in tissues^46^. Thus, the ability to perform a detailed study of vascular morphology and topology will potentially be a valuable method to evaluate the arteriogenic and angiogenic response to meniscal repair surgery on an area-by-area basis and then to determine which areas have more or less healing capacity. For example, using micro-CT, Duvall et al*.*^47^ found serial changes in the vascular network by analyzing morphological and topological vascular parameters, such as vessel volume, connectivity, number, and thickness that occur after an ischemic injury revealing that the body compensates by developing a dense network of smaller blood vessels to restore vascular volume. This process is observed through an aggregated evaluation of vascular structures. In another recent study^48^, the authors quantified the 3D vasculature and vascular parameters in the postnatal and adult mouse brain and found an increase with age in vascular volume fraction, capillary density, capillary branching, and tortuosity, and a decrease in diameter, segment length, and extravascular distance, highlighting the potential for a comprehensive analysis to study vascular topology. Also the degree of vascularization of the meniscus not merely depends on the location, but also on individual characteristics, such as age, presence of injuries and/or degenerative pathologies in the knee joint^6,16^. For this reason, future analyses could include a 3D investigation and quantification of the degree of vascularization of each area at different ages (adolescents, adults, and the elderly), and in the presence of meniscal injuries and pathologies that affect the structure and thus the function of the meniscus. Moreover, while our 3D imaging analysis provides new insights into the general anatomical structure and vascularity of the meniscus, it must be acknowledged that our technology is not applicable for in vivo use. Due to the inherent individual variability in meniscal vascularity, there is an urgent need to develop approaches that are both adaptive and personalized. Such advances will be critical in determining the continued relevance of the traditional classification and the one used in our study in the context of personalized medicine. In this regard, the ISAKOS Meniscal Documentation Subcommittee has recommended against the use of the traditional terms red-red, red-white and white-white due to their limitations in accurately reflecting the variability of vascular supply of the menisci^15^. Despite this, the older classification system remains widely accepted by surgeons and trainees, largely due to its simplicity and practicality^49^. This terminology still facilitates traditional understanding, alongside current recommendations and evolving guidelines.

This study is not without limitations. The sample size was limited, mainly due to the short availability of specimens and on the setting of the development of the perfusion process. The small sample size is a critical aspect that potentially affects the robustness and generalizability of our findings. However, the analysis suggests differences in vessel penetration between medial and lateral meniscus and in different circumferential and radial areas.

The human blood vessel lumen diameters range from approximately 25 mm for the aorta to 8 µm for the capillaries^50^, and that means that the smallest structures can be below our highest voxel resolution (15 μm). The voxel size of 15 μm was the highest nominal resolution achievable to keep our entire radial segments (specifically, the four quarter radial zones of Group C-high resolution) inside the field of view (FOV). This because, due to the cone-beam geometry of laboratory micro-CT systems, magnification is geometrically determined. Despite these resolution limitations, the imaging partial volume effect (PVE) presents a unique advantage in our context. In micro-CT imaging, a voxel's gray intensity value represents an average of the linear attenuation coefficients of all materials within its spatial domain^51^. Therefore, structures smaller than the voxel size can still influence the intensity value, particularly when there is a notable contrast between the structure and its surrounding tissue. In our study, this phenomenon allows for the potential identification of microvascular structures within the meniscus, even when their dimensions fall below the voxel size, due to the enhanced contrast provided by our imaging protocol.

Another limitation is the potential inhomogeneous perfusion and polymerization of the contrast agent for vascular visualization, resulting in interrupted vessel segments and incomplete vascular filling. To minimize the occurrence of these errors, we first restored the vascular network by injecting an inert dye solution prior to the contrast agent filling. In our study, we observed some discontinuities in the 3D visualization of microvascular structures, as shown in Figs. 4 and 5, where certain vessels appeared to be disconnected despite the meticulous perfusion of the contrast agent from the femoral artery. The perfusion protocol was rigorously designed, using a specialized pump to maintain a constant volume rate, with the goal of uniformly distributing the contrast agent throughout the vascular network. Despite these precautions, the formation of microbubbles could not be completely avoided, and their possible presence, due to insufficient time for their dissolution before perfusion, could contribute to the observed discontinuities. In addition, optimal perfusion requires a temperature range of 35–40 °C to maintain the appropriate viscosity of the contrast agent for effective distribution, especially in the finer capillary networks. However, given the nature of our specimens (whole human donor legs), we operated at room temperature, which may have affected the viscosity of the agent and consequently the quality of perfusion. The specimens were handled carefully to avoid any breakage that could introduce artifacts, although the risk of such events cannot be completely eliminated. The elasticity of the polymerized contrast agent provides some resistance to damage, but does not guarantee the absence of perfusion-related abnormalities.

Other factors that may contribute to the observed vessel discontinuities include aspects of our image processing and analysis methodology. Segmentation is a key process in the study. Ideal and universal micro-CT software tools for vascular image analysis and quantification are still lacking, and new algorithms and morphometric analysis that take into account the complex and tortuous nature of blood vessels should be developed and further implemented. In this study, an automatic segmentation was chosen to make the identification of blood vessels operator-independent. Compared to an operator-dependent segmentation, this type of approach minimized the variability of the results due to the subjective assessment of the evaluator, thus making the data obtained more valid, reliable and reproducible. However, despite the fact that our segmentation strategy aimed to capture all voxels containing the contrast agent, discontinuities may still manifest at the capillary level, where detection may depend on the partial volume effect.

In our study, the use of 70 kV to image the meniscus, although higher than the typical 30–60 kV range used for soft tissue imaging, was a deliberate choice aimed at focusing the output of the vascular contrast agent within the tissue. In addition, the fibrocartilaginous nature of the meniscus and the effects of chemical fixation did not prevent its visualization at this higher kV setting.

Due to the limitations imposed by Thiel fixation on staining affinity, traditional histological analysis was precluded for additional validation of our method. Although Thiel fixation preserves tissue flexibility and morphology for anatomical studies, it prevents the in-depth cellular and vascular examination compared to formalin^52,53^.

The main strength of this work is the 3D non-destructive visualization and quantification of blood vessels. Current approaches for 3D visualization, such as serial sectioning and vascular corrosion casting, are destructive, time-consuming and introduce artefacts. In contrast, micro-CT requires less time and the tissue is still suitable for further analysis, allowing the visualization of structures at different levels of organization, from large to small vessels.

In conclusion, our study represents an important step toward a comprehensive understanding of the meniscal microvasculature using advanced 3D visualization techniques through micro-CT. The primary objective of the research was to conduct exploratory data analysis aimed at identifying potential trends and patterns within our data set. Therefore, while our study offers preliminary observations and contributes to the existing body of knowledge, its limitations must be considered when interpreting the results. Future studies with expanded datasets are essential to increase the reliability of the findings and facilitate further understanding of the topic and its implications for clinical practice The insights acquired through this research, and future research using this approach, have the potential to impact the field of orthopedics by providing valuable knowledge regarding distribution of microvasculature that can lead to better therapeutic approaches.

## Data Availability

The data that support the findings of this study are available from the corresponding author upon reasonable request.
